# Crystal structure of chloramphenicol-metabolizing enzyme EstDL136 from a metagenome

**DOI:** 10.1371/journal.pone.0210298

**Published:** 2019-01-15

**Authors:** Sang-Hoon Kim, Pyeoung-Ann Kang, Keetae Han, Seon-Woo Lee, Sangkee Rhee

**Affiliations:** 1 Department of Agricultural Biotechnology, Seoul National University, Seoul, Korea; 2 Department of Applied Biology, Dong-A University, Busan, Korea; Karl-Franzens-Universitat Graz, AUSTRIA

## Abstract

Metagenomes often convey novel biological activities and therefore have gained considerable attention for use in biotechnological applications. Recently, metagenome-derived EstDL136 was found to possess chloramphenicol (Cm)-metabolizing features. Sequence analysis showed EstDL136 to be a member of the hormone-sensitive lipase (HSL) family with an Asp-His-Ser catalytic triad and a notable substrate specificity. In this study, we determined the crystal structures of EstDL136 and in a complex with Cm. Consistent with the high sequence similarity, the structure of EstDL136 is homologous to that of the HSL family. The active site of EstDL136 is a relatively shallow pocket that could accommodate Cm as a substrate as opposed to the long acyl chain substrates typical of the HSL family. Mutational analyses further suggested that several residues in the vicinity of the active site play roles in the Cm-binding of EstDL136. These results provide structural and functional insights into a metagenome-derived EstDL136.

## Introduction

Lipolytic enzymes hydrolyze triacylglycerols to produce fatty acids and glycerol [1]. Their presences are ubiquitous in most of living organisms including eukarya, bacteria and plant. Those lypolytic enzymes gain considerable attention for use in biotechnological applications from pharmaceutical and chemical industries [2,3]. Those enzymes are diverse, but can be classified by their substrate specificities: carboxylesterases (EC 3.1.1.1) and lipases (EC 3.1.1.3). In general, carboxylesterases hydrolyze partly water-soluble and small ester-containing molecules, whereas lipases exhibit their substrate specificity to water-insoluble long ester-containing molecules [4]. Alternatively, lipolytic enzymes are also classified by their amino acid sequence and the presence of conserved motifs. In this classification, enzymes can be divided into three groups; C, L and H [5,6]. The C group consists of cholinesterases and many other fungal lipases, and the L group includes lipo-protein lipases. The H group, also referred as hormone-sensitive lipases (HSL), represents the family of enzymes whose sequences are homologus with those of mammalian HSL [6,7]

HSLs commonly exhibit substrate promiscuity and the identification of HSLs with specific catalytic activities has been challenging. For this reason, metagenomic analyses, which often identify novel biological activities, have gained considerable attention. Recent studies using a metagenomic library from alluvial soil have characterized one enzyme related to the metabolism of antibiotics [8,9]. In sequence analysis, enzyme EstDL136 was found to be a member of the HSL family. EstDL136 exhibits two different activities for the catalytic metabolism of chloramphenicol (Cm), a widely used, broad-spectrum antibiotic; it acts as both a Cm acetate esterase (CAE) and a Cm hydrolase.

**Fig 1:**
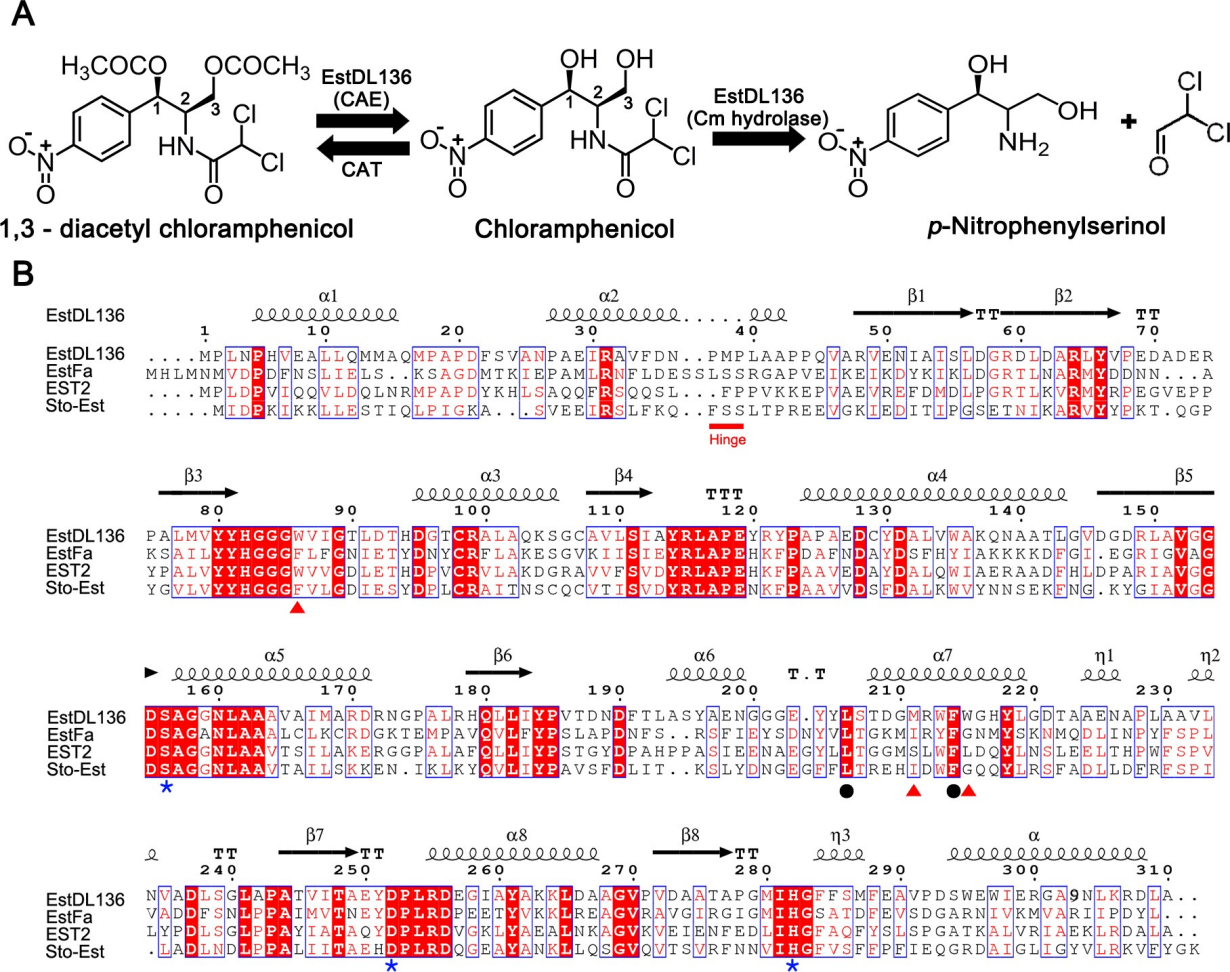
EstDL136 activity and sequence alignment with other HSLs. (A) The chemical structure of Cm is displayed. CAT activity allows acetylation of Cm at the C1 and C3 positions. The two different catalytic functions of EstDL136, its CAE and Cm hydrolase activities, are also illustrated. (B) The amino acid sequence of EstDL136 was compared with those of other HSLs by pairwise alignments. Three HSLs were selected for their different substrate specificities: EstFa (PDB code 3WJ2) for *p*-nitrophenyl-butyrate [27], EST2(M211S/R215L) mutant (PDB code 1QZ3) for *p*-nitrophenyl-hexanonate [26], and Sto-Est (PDB code 3AIK) for *p*-nitrophenyl-octanoate [28]. Highly conserved residues are shown in red and boxed in blue, whereas strictly conserved residues are displayed with a red background. Secondary structures are shown based on structural information from EstDL136(ΔPMP). Some residues that have possible roles in catalysis (blue asterisk), as possible determinants of the active site pocket (red triangles), and in the Cm-binding site (black circles) are indicated. This figure was prepared using ESPript [29].

As a CAE, EstDL136 de-acetylates the C1 and/or C3 position of 1,3-diacetyl Cm (Fig 1A) [8]. Cm is continually challenged by antibiotic resistance, and 1,3-diacetyl Cm is found in antibiotic resistance mechanisms. Among the many possible resistance mechanisms, bacterial Cm acetyltransferase (CAT)-dependent Cm inactivation is well characterized, in which CAT catalyzes acetylation of the hydroxyl group at the C1 and/or C3 position of Cm (Fig 1A). The resulting acetylated Cm no longer binds to its target ribosome and is unable to act as an antibiotic [10]. An enzyme with CAE activity could reverse CAT-dependent Cm inactivation. CAE activity has been reported in most Cm-producing *Streptomyces* spp., but identification of the responsible enzyme or its corresponding gene has been elusive [11–14]. EstDL136 also acts as a Cm hydrolase, directly affecting Cm metabolism and resistance. EstDL136 cleaves the amide linkage at C2 of Cm (Fig 1A) and thereby confers Cm resistance to bacteria heterologously expressing the EstDL136 gene [9].

Sequence analyses have indicated that metagenome-derived EstDL136 contains an Asp-His-Ser catalytic triad, a hallmark feature of the HSL family (Fig 1B), and it indeed shows high esterase activity toward *p*-nitrophenyl acetate, but a much lower activity toward *p*-nitrophenyl butyrate [8]. Therefore, EstDL136 exhibits relatively diverse activities, behaving as either a CAE or a Cm hydrolase. This study describes the crystal structures of EstDL136 and in a complex with a Cm, along with mutational analyses of active site residues. These results provide both structural and functional insights into the enzymatic activity of EstDL136.

## Materials and methods

### Protein expression and purification

Many variants of EstDL136 were constructed for crystallization, but only one formed a crystal suitable for structure determination. We therefore describe the expression, purification, and crystallization of that particular variant of EstDL136, in which three internal residues Pro37, Met38, and Pro39 were removed to facilitate crystallization. This strategy was suggested by the program Xtal Pred [15] and the resulting deletion mutant is referred to as EstDL136(ΔPMP). The gene encoding EstDL136 (NCBI Reference Sequence: HQ148666) was used as the template for PCR amplification, together with mutagenic primers. The resulting PCR product of EstDL136(ΔPMP) was introduced into the *Nde*I and *Xho*I sites of the pET-41b vector, producing the C-terminal His-tagged recombinant protein. EstDL136(ΔPMP) was expressed in *Escherichia coli* BL21 (DE3) cells (Merck) grown at 37°C in Luria-Bertani medium. Its expression was induced by the addition of 0.5 mM isopropyl-β-D-thiogalactopyranoside for 16 h at 20°C once the OD_600_ of the bacterial cells reached 0.6. Cells were harvested and sonicated in buffer A (50 mM Tris-HCl at pH 8.0, 500 mM NaCl, and 1 mM DTT), and the supernatant was obtained by centrifugation at 30,000 × *g* for 1 h at 4°C. The C-terminally His-tagged EstDL136(ΔPMP) was then purified using an immobilized metal affinity column (GE Healthcare) equilibrated with buffer A, followed by elution with buffer B (buffer A plus 500 mM imidazole). The eluted protein was dialyzed against buffer A and subjected to size-exclusion chromatography on a Superdex 200 column (GE Healthcare) that had been pre-equilibrated with buffer A.

For subsequent structural and functional studies, additional EstDL136 mutants were also constructed using the respective mutagenic primers, and the expression and purification of these mutants were essentially identical as those described above, unless specified otherwise. In particular, the EstDL136(ΔPMP/S156A) mutant, which contains an additional mutation at the proposed catalytic residue Ser156, was also used for structural studies of the complex formed between EstDL136 and Cm.

### Crystallization and data collection

C-terminal His-tagged EstDL136(ΔPMP) and EstDL136(ΔPMP/S156A) in buffer A were concentrated to 15 mg/ml. Crystallization of both enzymes was performed by the sitting-drop vapor-diffusion method at 295 K using a crystallization solution of 500 mM ammonium fluoride (pH 6.5), 30% PEG3350, 5% glycerol, and 120 mM TCEP. The binding of the Cm to EstDL136(ΔPMP/S156A) was achieved by soaking EstDL136(ΔPMP/S156A) crystals with 5 mM Cm (Sigma) in the crystallization solution for 50 min. Cryoprotection was performed using 20% (v/v) PEG400 for EstDL136(ΔPMP) and 3.5% (w/v) *myo*-inositol for EstDL136(ΔPMP/S156A).

X-ray diffraction data were collected at 100 K in the Pohang Accelerator Laboratory (Pohang, Korea). Data were processed using HKL2000 [16] and the details of the data collection are shown in Table 1.

**Table 1:** Data collection and refinement statistics.

Data set	EstDL136(ΔPMP)	EstDL136(ΔPMP/S156A)-Cm complex
PDB ID	6AAE	6IEY
**Data collection** Wavelength (Å)	0.97933	0.97933
Resolution (Å)	50.0–1.64 (1.70–1.64) ^a^	50.0–2.1 (2.18–2.10)
Unique reflections	100,067 (9,885)	46,963 (4,484)
Multiplicity	14.3 (13.1)	6.1 (5.6)
Completeness (%)	100 (100)	96.4 (94.1)
Mean I/sigma(I)	23.0 (1.2)	13.6 (2.1)
Wilson *B*-factors (Å^2^)	23.1	26.61
*R*-merge	0.11(0.12)	0.17(1.0)
CC_1/2_^b^	0.998(0.581)	0.995(0.543)
**Space group**	*P2*_*1*_*2*_*1*_*2*	*P2*_*1*_*2*_*1*_*2*
Unit cell *a*, *b*, *c* (Å)	118.9, 153.6, 44.2	118.5, 152.4, 44.1
α, β, γ (°)	90, 90, 90	90, 90, 90
**Refinement**		
*R*-work^c^	0.188 (0.290)	0.208 (0.261)
*R*-free^d^	0.216 (0.313)	0.256 (0.297)
No. of atoms		
Macromolecules	4704	4643
Ligands	187	20
Water	487	211
RMS(bonds) (Å)	0.007	0.008
RMS(angles) (°)	1.050	1.269
Ramachandran favored (%)	97.1	96.39
Ramachandran outliers (%)	0.0	0.49
Average *B*-factors (Å^2^)		
Macromolecules	28.50	30.54
Ligands	44.25	48.39
Water	37.32	33.83

^a^Numbers in parentheses refer to data in the highest resolution shell.

^b^The CC_1/2_ is the Pearson correlation coefficient (CC) calculated from each subset containing a random half of the measurements of unique reflection

^c^*R*_*work*_ = Σ ||*F*_obs_|-|*F*_cal_||/ Σ|*F*_obs_|

^d^*R*_*free*_ is the same as *R*_*obs*_ for a selected subset (5%) of the reflections that was not included in prior refinement calculations.

### Structure determination and refinement

Crystal of EstDL136(ΔPMP) and the EstDL136(ΔPMP/S156A)‒Cm complex both belong to the *P*2_1_2_1_2 space group with two monomers in the asymmetric unit. Their structures were solved by molecular replacement using PHENIX [17]. Specifically, the monomeric structure of carboxylesterase Est2 (PDB code 1U4N; sequence identity 45%) [18] was used as the search model for molecular replacement. Manual model building and refinement were performed using the COOT [19] and PHENIX programs [17], respectively, and the stereochemistry of the final model was evaluated using MolProbity [20]. When the structure of EstDL136(ΔPMP) was refined sufficiently, its monomeric structure was used as the search model for molecular replacement of the EstDL136(ΔPMP/S156A)‒Cm complex. Structural analyses were performed using programs in the CCP4 suite [21], and the figures presented in this study were generated using PyMol [22]. Details of the refinement are presented in Table 1.

### EstDL136 activity assays

The enzymatic activity of EstDL136 using *p*-nitrophenyl acetate as a substrate was assayed according to a previously published procedure [8]. Briefly, the esterase activity of EstDL136 was evaluated by monitoring an absorbance at 405 nm to detect the reaction product of *p*-nitrophenol.

For the activity assay, wild-type full-length and various mutant forms of EstDL136 were purified using an immobilized metal affinity column and a desalting column; 50 mM Tris (pH 8.0) buffer was used throughout the purification process. In the assay, the reaction mixture containing 50 mM Tris (pH 8.0) and ~2 nM EstDL136 was incubated at 30°C for 1 min, and *p*-nitrophenyl acetate at the given concentration was added to the reaction mixture. We then monitored changes in absorbance at 405 nm using a UV-visible spectrophotometer (Jasco, Japan). The initial velocity was determined between 40 and 70 s, and calculated as the *p*-nitrophenol concentration produced per minute. The molar extinction coefficient of *p*-nitrophenol at 405 nm is 16.8/mM cm, and *K*_m_ and *V*_max_ values were calculated using SigmaPlot (Systat Software).

## Results

### Structure of EstDL136(ΔPMP)

**Fig 2:**
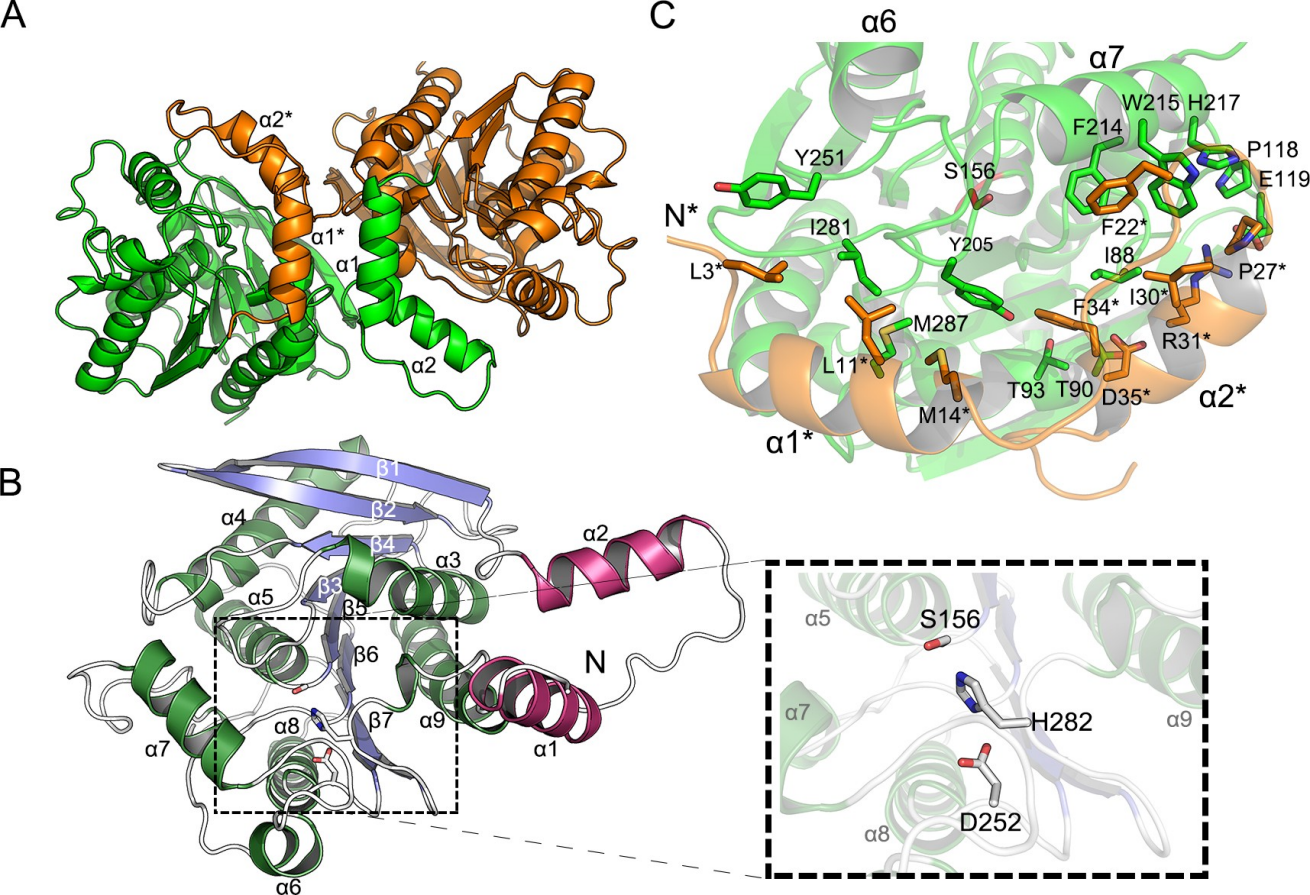
Dimeric and monomeric structures of EstDL136(ΔPMP). (A) The two monomers in the asymmetric unit of the apo-form EstDL136(ΔPMP) are shown in different colors. Note that the CAP domain of one monomer intercalates into the core α/β fold of the adjacent monomer. (B) The monomeric structure of the apo-form EstDL136(ΔPMP) is shown, with an enlarged view of the catalytic triad Asp252-His282-Ser156. Blue corresponds to the central β-sheet, green to α-helices, and magenta to the CAP region. (C) Intersubunit-interacting residues within 5Å are indicated for those in the CAP domain and an α/β fold of adjacent monomer. Note that the catalytic residue Ser156 is shown for the location of the active site.

A crystal of EstDL136(ΔPMP) contains two monomers per asymmetric unit (Fig 2A). Monomeric EstDL136(ΔPMP) consists of an N-terminal CAP domain and an α/β hydrolase domain (Fig 2B). The α/β hydrolase fold acts as a core structure in which the central eight β-strands form a concave β-sheet flanked by helices on both sides. Two helices, α3 and the C-terminal α9, are positioned on the concave side of the β-sheet with their helical axes almost parallel to the β-strands. Most of the remaining helices are on the convex surface of the central β-sheet. In addition to the core α/β fold, the 40-residue segment at the N-terminus forms a helix-loop-helix arrangement known as a CAP domain, in which α1 is almost orthogonal to α2 and protrudes from the main α/β fold. The putative catalytic triad, Asp252-His282-Ser156, is located at the C-terminal end of the central β-strands (Fig 2B).

**Fig 3:**
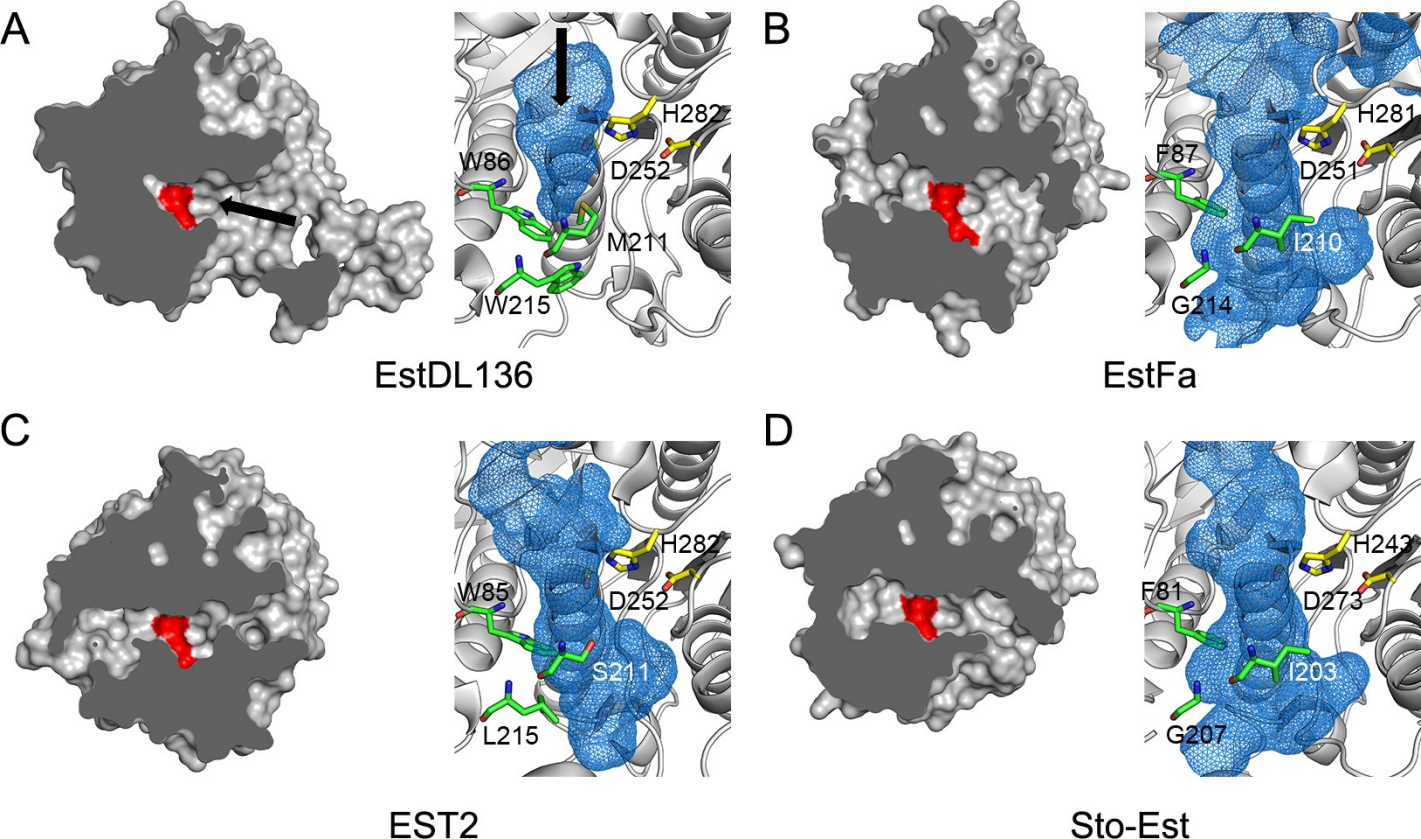
Active site of EstDL136 and other HSLs with different substrate specificities. (A‒D) EstDL136 (A) and three other HSLs from Fig 1B are shown: EstFa (B), EST2(M211S/R215L) (C), and Sto-Est (D). All four structures are superimposed and presented in an identical orientation. A surface representation of each enzyme is shown in the left panel. Catalytic residues are shown in red, and the active site entrance is indicated with an arrow. In the right panel, a potential position for the active site, calculated by the program PyMol, is indicated in blue, while catalytic histidine and aspartate residues are shown in yellow. Three residues, Trp86, Met211, and Trp215, occluding the active site pocket in EstDL136 are represented in green. Structurally equivalent residues in other HSLs are also indicated (see Fig 1B).

A structure similarity search using DALI [23] indicated that the overall structure and relative location of the catalytic triad in monomeric EstDL136(ΔPMP) are similar to those of other HSLs. Many HSL structures show Z-scores greater than 20, with EST2 (PDB ID: 1U4N) showing the highest Z-score of 43.6 [18]. Despite the high degree of structural similarity, there are noticeable differences in the size of the active site (Fig 3). EstDL136 has a relatively shallow, pocket-shaped active site, consistent with its substrate preference for *p*-nitrophenyl acetate over *p*-nitrophenyl butyrate [8]. Sequence and structural comparisons with other HSLs indicate that the active site size corresponds to its substrate preference (Fig 3). Consistent with a recent analysis in other HSLs [24], three residues in EstDL136 also appear to be major contributors in determining the active site size. Residues Trp86, Met211, and Trp215 are clustered at the bottom of the active site in EstDL136(ΔPMP) and possibly occlude the active site pocket (Fig 3A). In other HSLs, one or two of those residues are replaced with residues bearing a smaller side chain (Fig 1B). As a consequence, HSLs with a preference for substrates bearing long acyl chains exhibit more extended active sites (Fig 3B, 3C, and 3D)

Under our crystallization condition, the dimeric conformation of EstDL136(ΔPMP) is characterized. Two monomers in an asymmetric unit show a non-crystallographic, two-fold symmetry (Fig 2A). Interestingly, the CAP domains of the two monomers are in a domain-swap configuration in which the N-terminal helix α1 of the CAP domain almost intercalates into the potential vacancy between α1* and the main α/β fold of the adjacent monomer. Note that a superscript asterisk indicates the residue(s) or element(s) in the adjacent monomer. An omitted *Fo-Fc* map at the region connecting the CAP domain and the main α/β fold further validated a domain-swap configuration of the CAP domains (S1A Fig). As a result, the N-terminal helices α1 and α1* are packed in an anti-parallel orientation in the dimer (Fig 2A). Unlike the structural features of EstDL136(ΔPMP) as a dimer in crystalline state, size-exclusion chromatographic analyses indicated three different forms of EstDL136 are all present in solution as a monomer, including wild-type full-length EstDL136, EstDL136(ΔPMP), and EstDL136 in the absence of the CAP domain (Met1‒Asn36) (i.e., EstDL136(ΔCAP)) (S1B Fig). Therefore, there is no possibility that the deletion of a hinge region (i.e., ΔPMP) used in this study could cause the dimerization of EstDL136. Under these structural conditions, it is unlikely that the dimer observed in crystalline EstDL136(ΔPMP) represents a biologically relevant oligomeric conformation, but its structural features could still provide details on the monomeric enzyme with the active site residues.

### Structure of the EstDL136(ΔPMP/S156A) in a complex with Cm

**Fig 4:**
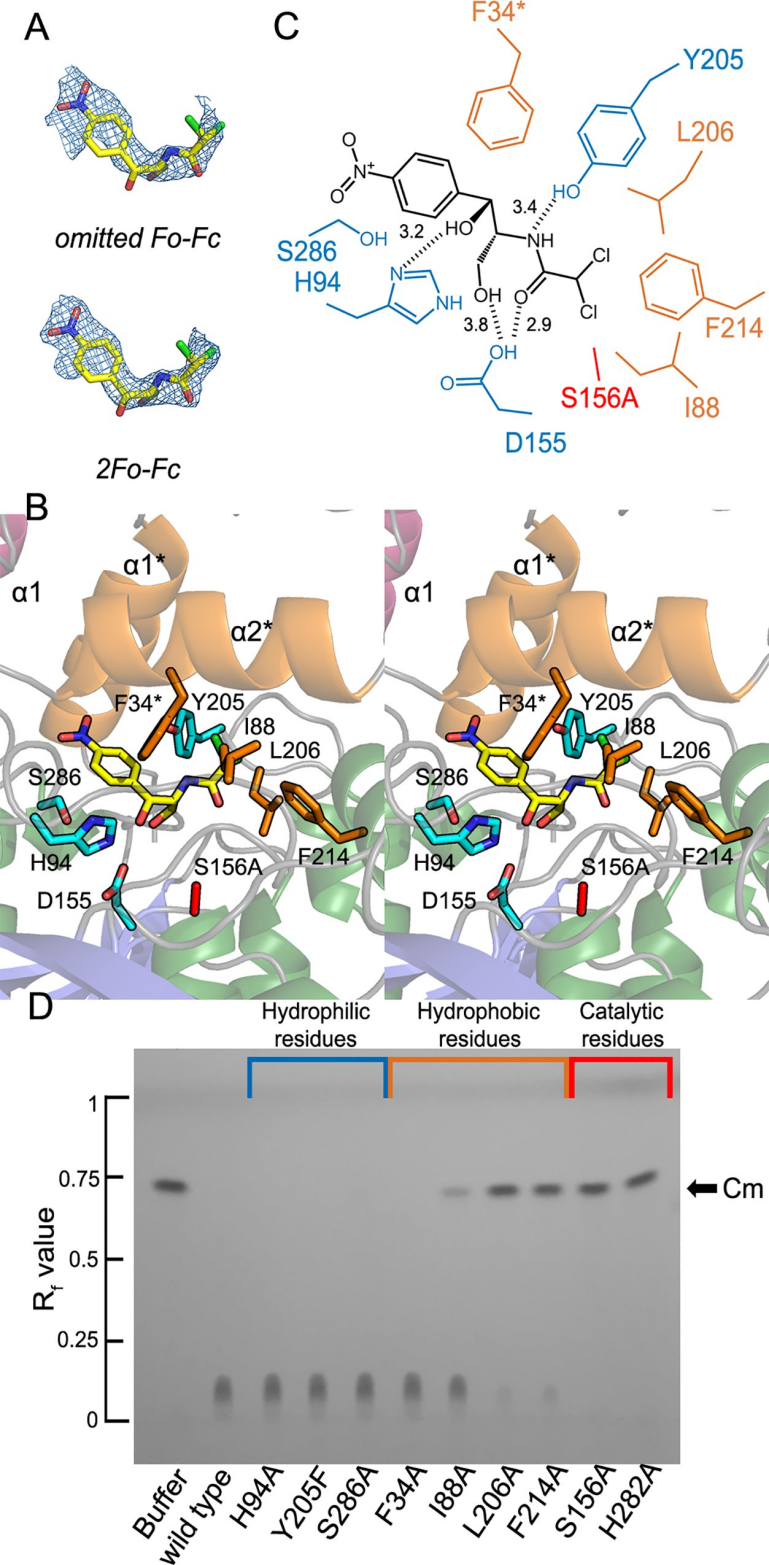
Binding of Cm in the active site of EstDL136(ΔPMP/S156A) and thin-layer chromatographic analysis of EstDL136 mutants. (A) The model of Cm is shown, with an electron density map. The overlaid map is an omitted *Fo-Fc* map at 2.0 σ in the upper panel and a *2Fo-Fc* map at 1.0 σ in the lower panel. (B) A stereo view of the active site of EstDL136(ΔPMP/S156A) is shown with bound Cm. The different colors, brown and cyan, are used to represent the hydrophobic and hydrophilic Cm-interacting residues, respectively. Note that α1* and α2* in the CAP domain is from the adjacent monomer, and that the catalytic Ser156 residue has been replaced with alanine. (C) A schematic representation shows the binding mode of Cm in the active site of EstDL136(ΔPMP/S156A) as shown in Fig 4B. (D) Thin-layer chromatographic analyses of various EstDL136 derivatives are shown. Analyses were carried out, using wild-type full-length and various mutant forms of EstDL136, according to a previously published procedure [8]. Various mutants of EstDL136 (25 μM) were incubated for 2 h at 30°C in 4.25 mM Cm, and the resulting mixture was applied to glass plates coated with silica gel 60 F254 (Merck) and developed using an acetone:chloroform ratio of 15:85. Nine mutant enzymes were prepared: F34A, I88A, H94A, S156A, Y205F, L206A, F214A, H282A, and S286A.

The binding of Cm to EstDL136 was achieved using the EstDL136(ΔPMP/S156A) mutant (Fig 4A). The EstDL136(ΔPMP/S156A)‒Cm crystal also contains two monomers in the asymmetric unit. In the dimer, each monomer adopts a conformation essentially identical to that observed in EstDL136(ΔPMP), with a root-mean-square-deviation of 0.91 Å for 300 Cα atoms. Unlike other HSLs, which show ligand-induced conformational changes in the CAP region, the CAP domain of the EstDL136(ΔPMP)‒Cm complex remained identical to that of the unliganded form. These structural behaviors could arise from extensive interactions between two monomers and/or mutation we introduced. Previous structural analyses of HSLs suggest that residues corresponding to the tri-peptide Pro37-Met38-Pro39 in EstDL136, which was removed for crystallization, play an important role as the hinge region in an open-to-close conformational transition of the CAP region [18]. There are also extensive intersubunit interactions between the CAP domain and the α/β fold of the adjacent monomer (Fig 2A–2C). The PISA analysis revealed that those intersubunit interactions include more than 100 hydrophobic interactions, 22 hydrogen bonds, and 11 salt bridges [25]. Therefore, in addition to deletion of the potential hinge region in EstDL136(ΔPMP/S156A), it could be possible that extensive intersubunit interactions affect conformational mobility of the CAP region.

Cm was bound to the active site of the EstDL136(ΔPMP/S156A)‒Cm complex (Fig 4B). Specifically, the *p-nitro* group of Cm is located near the entrance to the active site pocket, and its C2 group points toward Ala156 located at the bottom of the pocket, a mutated residue for the catalytic residue Ser156. There are no obvious stacking interactions between the aromatic ring of Cm and the active site residues. However, the presence of bulky side chains in the active site, and in the CAP domain, appears to dictate the binding mode of Cm. In particular, the CAP domain of the adjacent monomer interacts with bound Cm (Fig 4B and 4C). Residues within 5.0 Å of the Cm-binding site include Phe34*, Ile88, His94, Asp155, Tyr205, Leu206, Phe214, and Ser286.

We then performed mutational analyses of these active site residues using wild-type full-length EstDL136 as a template. First, residues within 5.0 Å of Cm (Fig 4B and 4C), which presumably interact with Cm, were selected for mutagenesis, producing F34A, I88A, H94A, Y205F, L206A, F214A, and S286A. Mutations of the catalytic triad were also constructed, including S156A and H282A. Mutations of Asp252 and Asp155 were also constructed, but mutants such as D252A, D252N, and D155A were expressed as inclusion bodies. The catalytic activity of each mutant was qualitatively evaluated using thin-layer chromatography with Cm as the substrate (Fig 4D). Most mutants were still active, but L206A, F214A, S156A, and H282A were inactive. The Ser156 and His282 residues are members of the catalytic triad, and thus the catalytic inefficacy of mutants S156A and H282A is readily justified. Therefore, hydrophobic interactions mediated by Leu206 and Phe214 appear to play a role in the activity of EstDL136.

Further kinetic analyses of L206A and F214A were carried out using *p*-nitrophenyl acetate as the substrate (Table 2 and S2 Fig). Three- and four-fold increases in *K*_*m*_ were observed for the L206A and F214A mutants, respectively, while their *k*_*cat*_ values were similar to that of the wild-type full-length enzyme. Those changes resulted in enzyme efficiencies, defined by *k*_*cat*_/*K*_*m*_, of 31% and 26% that of the wild-type full-length enzyme. Consistent with our structural analyses, those mutations largely affected the binding efficiency of the substrate.

**Table 2:** Kinetic parameters of EstDL136 and its mutants.

	*K*_m_ (mM)	*k*_cat_ (sec^-1^)	*k*_cat_ */K*_m_ (sec^-1^ mM^-1^)
WT	0.14 (0.016)^a^	622 (19.9)	4.4 × 10^3^
L206A	0.40 (0.030)	547 (14.3)	1.4 × 10^3^
F214A	0.55 (0.051)	641 (22.2)	1.2 × 10^3^

^a^Values in parentheses are standard errors estimated by the program SigmaPlot.

## Discussion

From sequence perspective, EstDL136 is a member of the HSL family, with a Cm-metabolizing activity. Structural analyses in this study have validated that EstDL136 contains structural features characteristic of the HSL family, with an α/β hydrolase fold and a CAP domain. We also found that the active site of EstDL136 is relatively shallow and able to accommodate Cm instead of substrates bearing long acyl chains, which are preferred more typically by HSLs (Fig 3).

A closer view on the Cm-binding mode were characterized in a structure of the EstDL136(ΔPMP/S156A)‒Cm complex, along with mutational analyses. We found that Leu206 and Phe214 play a role in Cm binding to the active site (Fig 4B–4D and Table 2). Based on locations of those residues near the C2 group of Cm, Leu206 and Phe214 possibly facilitate a correct orientation of the C2 group toward a catalytic serine residue. We, however, rule out that Leu206 and Phe214 are structural elements essential for the Cm substrate specificity, given that two residues are conserved in the HSL family (Fig 1B), suggesting that those residues play a general or common role in the binding of substrates to the active site of the HSL family (Table 2). Unfortunately, in this study we could not identify structural elements dictating the Cm substrate specificity for the matagenome-originated EstDL136. However, from structural and sequence perspective, Trp86, Met211, and Trp215 are possible candidates for the Cm substrate specificity, given their sequence diversities among the HSL family (Fig 1B) and their involvements in forming the active site pocket (Fig 3A). In the previous studies [24, 26], replacement of the active site residues with smaller-chain residues resulted in significant changes in substrate specificity. Interestingly, the mutated residues in these studies were localized to the vicinity of the three residues, i.e., Trp86, Met211, and Trp215 in this study (Figs 1B and 3). Further studies of those residues will provide details of the substrate specificity for the matagenome-originated EstDL136.

## Supporting information

S1 Fig Domain-swap configuration of the CAP domain and size-exclusion chromatographic analyses of EstDL136. (A) An omitted *Fo-Fc* electron map contoured at 2.5 σ is shown for the region (i.e., Leu 40 to Ala 47) connecting the CAP domain and the α/β hydrolase domain. These observations further validate a domain-swap configuration of the CAP domain observed in this study. (B) Size-exclusion chromatographic analyses are shown for the wild-type full-length EstDL136 (black), EstDL136(ΔCAP) (red) and EstDL136(ΔPMP) (green). Each protein, in the presence 10 mM PBS plus 500 mM NaCl, was eluted on a Superdex 200 column (GE Healthcare). Chromatograms were compared against 12‒200 kDa molecular mass markers (Sigma Chemical). Note that an eluted peak of all three proteins corresponds to 29.2 kDa of monomer.
(TIF)

S2 Fig Steady-state kinetic analysis of EstDL136 enzymes. *K*_m_ and *k*_*cat*_ values were calculated using SigmaPlot (Systat Software). Enzyme assays were carried out as described in Section 2.4.
(TIF)
