# Supplementary figures and images for: Crystal structure of chloramphenicol-metabolizing enzyme EstDL136 from a metagenome
Source: PLoS One. 2019 Jan 15;14(1):e0210298. doi: 10.1371/journal.pone.0210298 (PMC6333409; doi:10.1371/journal.pone.0210298)

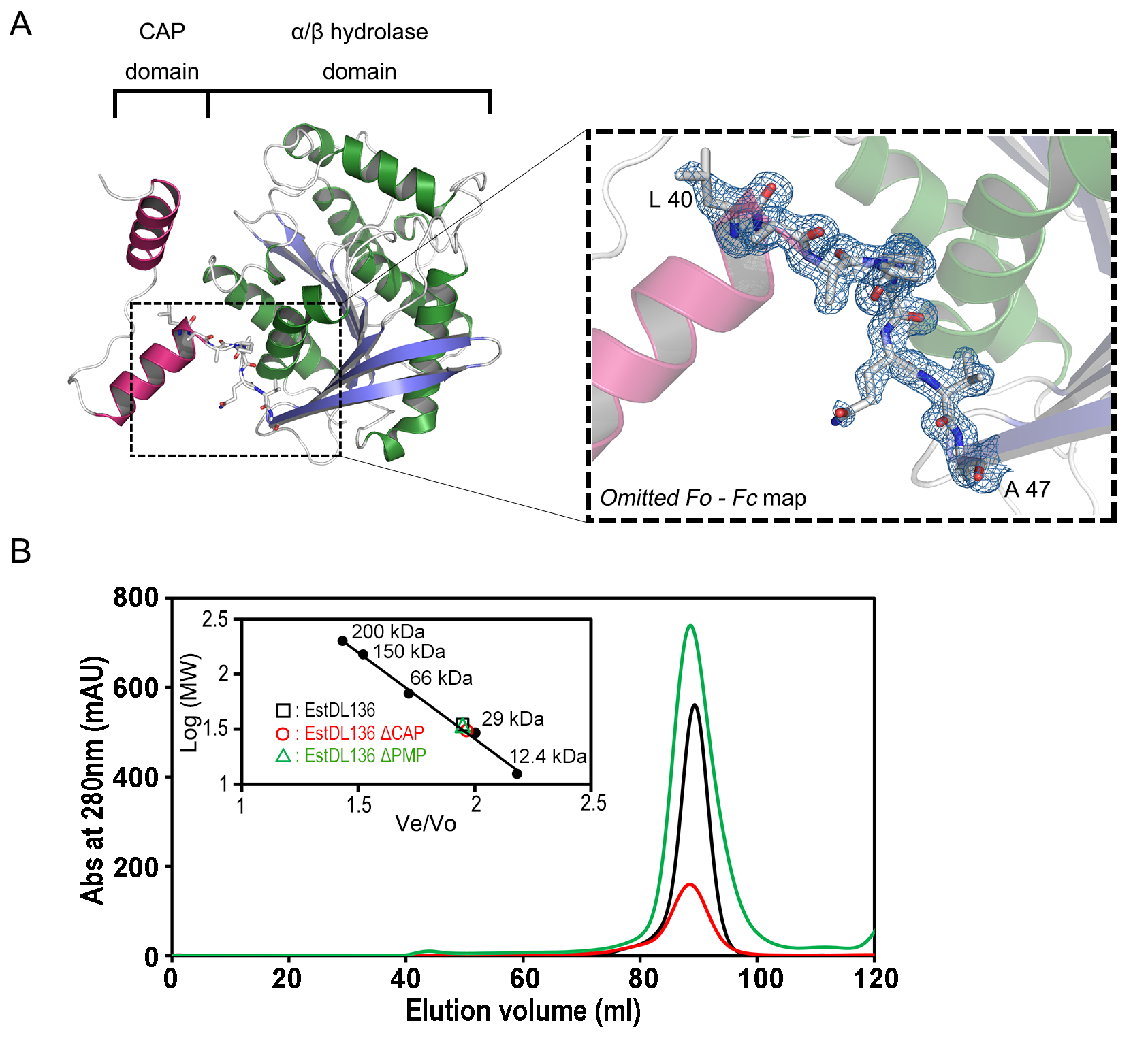

Supplement: S1 Fig — (A) An omitted Fo-Fc electron map contoured at 2.5 σ is shown for the region (i.e., Leu 40 to Ala 47) connecting the CAP domain and the α/β hydrolase domain. These observations further validate a domain-swap configuration of the CAP domain observed in this study. (B) Size-exclusion chromatographic analyses are shown for the wild-type full-length EstDL136 (black), EstDL136(ΔCAP) (red) and EstDL136(ΔPMP) (green). Each protein, in the presence 10 mM PBS plus 500 mM NaCl, was eluted on a Superdex 200 column (GE Healthcare). Chromatograms were compared against 12‒200 kDa molecular mass markers (Sigma Chemical). Note that an eluted peak of all three proteins corresponds to 29.2 kDa of monomer. (TIF) [file pone.0210298.s001.tif]

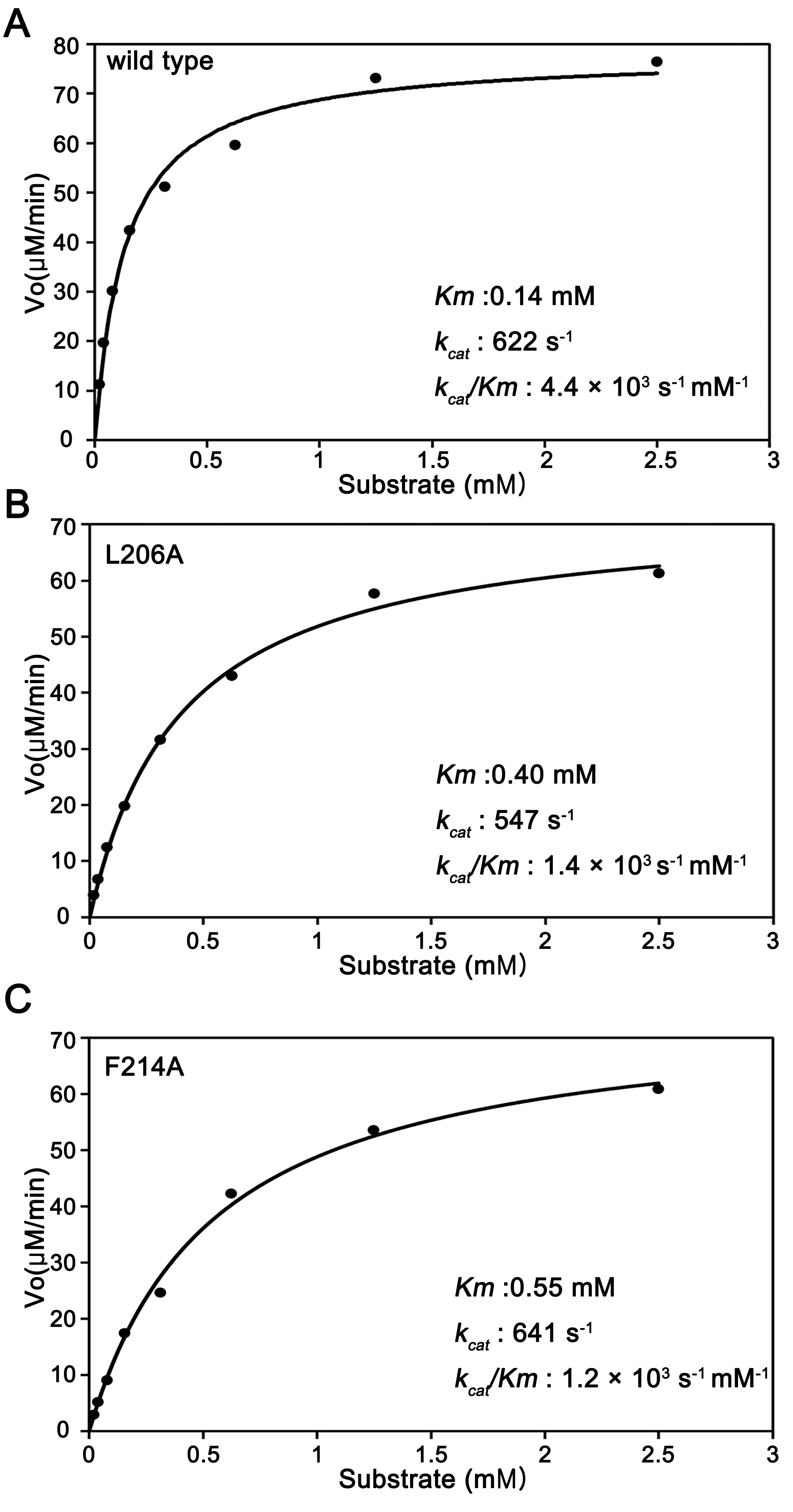

Supplement: S2 Fig — Km and kcat values were calculated using SigmaPlot (Systat Software). Enzyme assays were carried out as described in Section 2.4. (TIF) [file pone.0210298.s002.tif]
